# Prenatal opioid exposure and the early life epigenome: results from ECHO

**DOI:** 10.1080/14659891.2024.2356569

**Published:** 2024-05-27

**Authors:** Rose Schrott, Henri Garrison-Desany, Lyndsay Avalos, Carrie V. Breton, Dana M. Dabelea, Karen Derefinko, Anne Dunlop, Fang Fang, Abigail Gaylord, Torie Grant, Marie-France Hivert, Margaret R. Karagas, Anna K. Knight, Barry Lester, Kristen Lyall, Cindy McEvoy, Ruby Nguyen, Grier Page, Alison Paquette, Douglas Ruden, Lyndsey E Shorey-Kendrick, Alicia K. Smith, Eliot Spindel, Heather E. Volk, Christine Ladd-Acosta

**Affiliations:** a Wendy Klag Center for Autism and Developmental Disabilities, Johns Hopkins Bloomberg School of Public Health, Baltimore, Maryland, USA; b Department of Mental Health, Johns Hopkins Bloomberg School of Public Health, Baltimore, Maryland, USA; c Department of Social and Behavioral Sciences, Harvard T. H. Chan School of Public Health, Boston, Massachusetts, United States; d Division of Research, Kaiser Permanente Northern California, Oakland, California, USA; e Department of Preventive Medicine, Keck School of Medicine, University of Southern California, Los Angeles, California, USA; f Lifecourse Epidemiology of Adiposity and Diabetes Center, University of Colorado Anschutz Medical Campus, Aurora, Colorado, USA; g Department of Preventive Medicine and Department of Pharmacology, Addiction Science, and Toxicology, The University of Tennessee Health Science Center, Memphis, Tennessee, USA; h Department of Gynecology and Obstetrics, Emory University School of Medicine, Atlanta, Georgia; i RTI International, Research Triangle Park, Durham, North Carolina, USA;; j Department of Environmental Health Sciences, Columbia Mailman School of Public Health, New York, New York, USA; k Department of Medicine and Department of Pediatrics, Johns Hopkins University School of Medicine, Baltimore,United States;; l Department of Population Medicine, Harvard Medical School and Harvard Pilgrim Health Care Institute, Boston, Massachusetts, USA; m Department of Epidemiology, Geisel School of Medicine, Dartmouth College, Hanover, New Hampshire, USA; n Departments of Psychiatry and Pediatrics, Alpert Medical School of Brown University, Providence, Rhode Island, USA; o AJ Drexel Autism Institute, Drexel University, Philadelphia, Pennsylvania, USA; p Department of Pediatrics, Oregon Health and Science University, Portland, Oregon, USA; q Division of Epidemiology and Community Health, University of Minnesota, Minneapolis, Minnesota, USA; r Department of Pediatrics, School of Medicine, University of Washington, Seattle, Washington, USA;; s Department of Obstetrics and Gynecology, Wayne State University, Detroit, Michigan, United States;; t Division of Neuroscience, Oregon National Primate Research Center, Oregon Health & Science University, Beaverton, Oregon, United States; u Department of Epidemiology, Johns Hopkins Bloomberg School of Public Health, Baltimore, Maryland, USA

**Keywords:** DNA methylation, opioids, prenatal, epigenetics

## Abstract

**Background:**

Prenatal opioid exposure has been associated with adverse child health outcomes. Changes to the epigenome provide a plausible mechanism through which effects may be elicited. We investigated whether prenatal opioid exposure was associated with locus-specific changes in umbilical cord blood DNA methylation (DNAm) and gestational epigenetic age.

**Methods:**

We leveraged data from the Environmental influences on Child Health Outcomes cohort. Prenatal opioid data was obtained from maternal self-report and/or medical record data. DNAm measures were generated from blood biospecimens collected at birth. Linear regression models tested associations between prenatal maternal opioid exposure and epigenetic outcomes in crude and fully adjusted models.

**Results:**

We tested the association between prenatal opioid exposure and cord blood DNAm at 15 CpG sites in 385 (*n* = 25 exposed, *n* = 360 unexposed) individuals from three cohorts. We identified a single CpG site (cg14303187) that was nominally associated with prenatal opioid exposure (*p* = 0.02, β = −0.012; 95% CI, −0.023 to −0.0012). No significant associations between exposure and gestational epigenetic age were found (*n* = 716 individuals from eight cohorts; *n* = 29 exposed, *n* = 687 unexposed).

**Conclusions:**

We identified a nominally significant association between prenatal opioid exposure and DNAm at one CpG site. Future studies should continue investigating the effect of this exposure on the epigenome.

## Introduction

There has been a stark increase in the rate of opioid use disorder among pregnant people (Mactier & Hamilton, 2020; Weller et al., 2021). More specifically, the number of pregnant people with opioid-related diagnoses at delivery increased by 131% from 2010 to 2017 (Hirai et al., 2021). Similarly, rates of babies born with neonatal opioid withdrawal syndrome (NOWS) diagnoses in the US have increased in recent years (Conradt et al., 2019; Hirai et al., 2021). Babies experiencing NOWS present a distinct set of symptoms, including high-pitched crying, irritability, and difficulty sleeping (Hirai et al., 2021; Weller et al., 2021). However, not all babies with prenatal opioid exposure experience NOWS, and symptom severity can vary among infants. There are gaps in knowledge surrounding the lasting impact of prenatal opioid exposure on a child’s development, and the biological mechanisms involved in the resulting symptoms are not well characterized. This research study aims to begin to address these gaps.

Changes to the offspring DNA methylome provide one plausible mechanism whereby prenatal exposures might impact later life health and disease. DNA methylation changes are responsive to the environment (Marsit, 2015) and play critical roles in regulating early life development through epigenetic reprogramming and patterning of the gametic, placental, and fetal landscapes (Koukoura et al., 2012; Zeng & Chen, 2019). Few studies have investigated the effect of prenatal exposure to opioids on the DNA methylome. Of those that have, a small number have taken an epigenome-wide approach (Borrelli et al., 2022; Henri Garrison-Desany et al., 2022; Radhakrishna et al., 2021), while others have primarily focused on the analysis of DNA methylation changes in candidate genes such as the mu-opioid receptor gene (*OPRM1*) in cord blood (Wachman et al., 2014) and placenta (Wachman et al., 2020). It is crucial to better understand the mechanisms through which prenatal opioid exposure impacts offspring health and development as rates of use during pregnancy climb.

Epigenetic age, partially reflecting biological aging due to environmental factors, has emerged as a marker of an individual’s overall health (Horvath, 2013; Ryan, 2021). Tools to measure epigenetic age have recently gained interest in the field of environmental health to better understand how certain exposures might be associated with accelerated or decelerated epigenetic aging relative to chronological age. Accelerated epigenetic aging has been associated with numerous diseases, while decelerated epigenetic aging is associated with improved health outcomes in adults (Horvath, 2013; Ryan, 2021). The recent development of an epigenetic gestational age clock allows us to ask important questions about how the early life environment might impact a child’s developmental epigenetic age at birth (Knight et al., 2016). For example, a recent study implementing the gestational epigenetic age clocks demonstrated an association between prenatal exposure to ambient air pollution and decelerated epigenetic age at birth in newborns, which is believed to be reflective of a preterm-birth state (Song et al., 2022). Existing literature demonstrates associations between prenatal opioid use and adverse health outcomes in babies, including being born preterm, though the underlying mechanisms are not well established (Interrante et al., 2021). Thus, it is critical to understand whether gestational epigenetic age might be influenced by this prenatal exposure, as this could shed light on the affected biological mechanisms.

Here, we sought to expand upon the current literature through leveraging existing data and resources from the Environmental influences on Child Health Outcomes (ECHO) cohort. We first analyzed cord blood methylation at candidate CpG sites initially detected in a previously published Boston Birth Cohort (BBC) study (Henri Garrison-Desany et al., 2022) in an effort to replicate distinct loci whose cord blood DNA methylation is altered following this same exposure in ECHO. We then investigated the relationship between prenatal opioid exposure and biological age at birth through implementation of, and analysis with, the Knight gestational age epigenetic clock algorithm (Knight et al., 2016).

## Methods

### Study population.

The ECHO program is a multisite national program of more than 60 observational cohorts that is dedicated to understanding the role of the environment in child health and development (Blackwell et al., 2018; Bush et al., 2020; Jacobson et al., 2018). To test our hypotheses, we applied the following inclusion criteria: (1) participants must have high-quality child DNA methylation data available on an Illumina platform that was collected from cord blood or dried blood spots, and (2) participants must have data collected on maternal opioid use during pregnancy. This cohort study used data available through the ECHO data repository as of the August 31 2022 data lock date. The study protocol was approved by the local or single ECHO institutional review board.

### Definition of maternal opioid use and covariates.

Prenatal opioid use was broadly defined as any prenatal opioid use, regardless of whether prescription and/or recreational use status was known. Data were obtained from self-report or medical record abstraction. Opioid medications given during labor and delivery were not considered prenatal opioid use for the purposes of this analysis nor were opioid medications for dental procedures and toothache or cough syrup with codeine. Additional covariate data including maternal age at delivery, self-reported maternal race and ethnicity, maternal education, maternal alcohol, tobacco, and marijuana use during pregnancy, maternal depression history, gestational age (for locus-specific analyses), and child sex were obtained from participant report on sociodemographic questionnaires or via medical records. We included race and ethnicity as covariates in this study because of concerns that each could independently associate with both prenatal opioid use and DNA methylation or epigenetic age, and we believed that they were unlikely to mediate the exposure–outcome relationship based on available evidence. Chronological gestational age at birth, in weeks, was calculated from the last menstrual period or from prenatal ultrasonography examination.

### Biospecimen collection and DNA methylation data measures.

Cohorts included in the present study collected cord blood or newborn blood spots through approved site-specific protocols. DNA methylation was measured using the Infinium HumanMethylation27 (27K) array, the Infinium HumanMethylation450 (450K) array, or the Infinium Methylation EPIC (EPIC) array. Raw.IDAT files from each cohort were imported into the minfi processing pipeline in R to calculate beta values for each probe and sample, and beta values (estimating the degree of DNA methylation from 0 to 1) were calculated as previously described (Ladd-Acosta et al., 2023). Sample and probe-level filters were applied to the data as previously described (Ladd-Acosta et al., 2023). Background correction and normalization were performed on the filtered data sets for each methylation platform using the noob function in *minfi* as described (Ladd-Acosta et al., 2023). Finally, additional sample filters were applied to the noob-corrected data sets based on age and tissue-type discrepancies to achieve clean data sets for each platform (Ladd-Acosta et al., 2023). Clean data sets were used as input for site-specific DNAm and gestational epigenetic age analyses, and cell composition estimations were used to permit adjustment for cell type.

### Site-specific CpG analyses.

After data processing there were 385 individuals from three cohorts (the New Hampshire Birth Cohort Study, the Early Autism Risk Longitudinal Investigation (EARLI), and Project Viva; Supplementary Figure 1 (left)) with cord blood DNA methylation data from the 450K platform and maternal opioid use data. Participants for this analysis were born between 1999–2012 and were recruited from study sites in New Hampshire, Maryland, Pennsylvania, California, and Massachusetts. We selected 15 CpG sites from a previously published BBC epigenome-wide association study looking at prenatal opioid exposure and cord blood DNA methylation at birth on the EPIC array (Henri Garrison-Desany et al., 2022) to be investigated in this study. We limited our analysis to just 15 CpG sites to balance statistical power, given our sample size, with examination of high priority loci that were previously identified in a specific US subpopulation from the metro-Boston region (BBC study). Our criteria for CpG inclusion in this study were: 1) present on the 450K array and passed QC filters in ECHO; 2) reached statistical significance in the BBC dataset; and 3) had the largest magnitude of methylation difference in the BBC dataset to optimize our chances of being able to discover CpGs where methylation robustly showed changes related to opioid exposure in this broader US population sample.

### Gestational epigenetic age calculations.

After data processing, there were 716 individuals from eight cohorts (the Atlanta ECHO Cohort of Emory University, MADRES, the New Hampshire Birth Cohort Study, CANDLE, EARLI, Project Viva, ARCH, and the NYU Children’s Health and Environment Study; Supplementary Figure 1 (right)) with gestational epigenetic age measures from cord blood and dried blood spot DNA methylation measures at birth and maternal opioid use data. Participants in this analysis were born between 1999 and 2019 and were recruited from study sites in Georgia, California, New Hampshire, Tennessee, Maryland, Pennsylvania, Massachusetts, Michigan, and New York. We chose to use the Knight gestational epigenetic clock based on its robust previous performance in ECHO, the ability to easily use data from multiple Illumina array platforms as measurements from the 27K, 450K, and EPIC arrays were included, and the wide range of diverse gestational ages included (Knight et al., 2016). Extrinsic age acceleration (EAA) was calculated using a linear regression model with chronological gestational age at birth (in completed weeks) as the independent variable and epigenetic age as the dependent variable. Extracted residuals from the linear regression model represent individuals’ EAA.

Cord blood and blood spot intrinsic age acceleration (IAA) were calculated separately for each tissue type. A linear regression model was used with chronological gestational age at birth as the independent variable and gestational epigenetic age as the dependent variable, adjusted for tissue-type-specific estimated cell proportions. The estimateCellCounts() function from the *minfi* Bioconductor package was used to generate cell composition estimates, and the compositeCellType argument was set to “blood” or “cord blood” for the respective tissue types. Cell types for peripheral blood spot samples included granulocytes, monocytes, natural killer cells, B cells, CD4 cells, and CD8 cells. For cord blood samples, the cell types accounted for include granulocytes, monocytes, natural killer cells, B cells, nucleated red blood cells, CD4 cells, and CD8 cells. The residuals extracted from the models represent individuals’ IAAs.

### Statistical analyses.

Data analysis was performed from September 2022 to March 2023. All data analyses were performed in R statistical software. Maternal opioid use and all covariates of interest listed above were summarized with descriptive statistics, with frequency, percentage, and a chi-square test for independent p-values reported for categorical variables, and mean [SD] or median, and a two-sample t-test p-value reported for continuous and numeric variables. Linear regression models were fitted with prenatal maternal opioid use as the exposure, and CpG-specific DNA methylation, EAA, and IAA were tested independently as outcomes in crude and fully adjusted models. Fully adjusted models included the covariates mentioned above, as well as batch ID, site ID, and estimated cell-type proportions. Point estimates, 95% CIs, and p-values are reported, with a 2-sided *p* < 0.05 taken to be a nominally significant association between opioid exposure and the outcome. To handle missing data, we performed multiple imputations using chained equations (MICE) for each incomplete variable as long as the missingness was <30%, using the *mice* R package developed by Stef van Buuren. We used predictive mean matching for numeric variables (maternal depression); logistic regression for dichotomous variables (maternal ethnicity; maternal tobacco use; maternal alcohol use; maternal marijuana use); polytomous regression for unordered categorical variables (maternal race); and proportional odds for ordered categorical variables (maternal education). Following imputation, we fit linear regression models to the 10 imputed data sets and then pooled the results following Rubin’s rules to account for any uncertainty. We took cohort differences into consideration during imputation to uphold the missing at random assumption.

## Results

### Study demographics.

Focusing first on the CpG site-specific analytic population, there were 25 individuals whose mothers used opioids during pregnancy and 360 individuals whose did not. Participants were similar across groups for all demographics (Table 1). The mean maternal age of the mothers who used opioids was slightly older than those who did not (34.6 years compared to 32.4 years, respectively; *p* = .06). Consumption of other substances such as alcohol, tobacco, and marijuana during pregnancy was low across both groups, and most mothers were non-Hispanic white (Table 1). Most mothers had some college education for both exposed and unexposed groups, and there were no differences in maternal depression between groups. There were no differences in child sex or gestational age in the exposure group (Table 1).

Of the 716 participants included in the epigenetic age analysis, 29 had prenatal exposure to opioids and 687 did not (Table 2). No statistically significant differences in demographics were detected across exposed and unexposed groups (Table 2). As with the CpG-site specific analytic population, mothers who used opioids were slightly older compared to those who did not (33.1 years compared to 30.8 years, respectively; *p* = .06). Use of alcohol, tobacco, or marijuana products during pregnancy was low for both groups. Most mothers were white, though fewer mothers self-identified as white in the unexposed group compared to the exposed group, and most were non-Hispanic and had some college for both exposed and unexposed groups. There were no differences in maternal depression between groups nor were there differences in child sex or gestational age (Table 2).

### CpG-site analyses.

The mean methylation difference at each of the CpG sites analyzed is reported in Table 3, and the absolute methylation differences range from 0.00% to 1.30% methylation change. Of these, the majority (*n* = 10) of CpG sites were hypermethylated in cord blood from infants whose mothers used opioids during pregnancy compared to those who did not (Table 3). Overall, the magnitude of methylation difference was smaller in ECHO than in the comparison BBC study.

We performed crude and fully adjusted linear regression models to determine the association between prenatal opioid exposure and cord blood DNAm at each of the CpG sites of interest. Fully adjusted models included the covariates mentioned above. A covariate correlation plot demonstrated no collinearity between covariates (Suplementary Figure 2). We found that prenatal opioid exposure was nominally significantly associated with DNAm at CpG site cg14303187, which is annotated to the SHC Adaptor Protein 3 (*SHC3*) gene (*p* = .02, β = −0.012; 95% CI, −0.023 to −0.0012; Table 4 and full results in Supplementary Table 1). There were no CpG sites that remained significant following the Bonferroni correction for the 15 CpG sites analyzed (*p* = .003).

### Epigenetic age analyses.

We next examined whether prenatal opioid exposure was associated with gestational epigenetic age acceleration or deceleration, where acceleration and deceleration were defined as the positive or negative residuals taken from regressing epigenetic age on chronologic age, respectively. A covariate correlation plot demonstrated no collinearity between covariates (Suplementary Figure 3). Crude and fully adjusted linear regression models (including covariates listed above) for EAA and IAA were run, and no significant associations between prenatal opioid exposure and either EAA or IAA at birth were identified (Table 5, Supplementary Figure 4).

### Discussion.

The present study had two primary objectives. The first objective was to determine whether DNA methylation changes resulting from prenatal opioid exposure at birth – identified in cord blood from an independent study – were similarly detectable in cord blood tissues collected at birth in ECHO. With this first objective, we sought to confirm these previously reported methylation changes in an independent cohort using the ECHO study. Here we identified a single CpG site, cg14303187, whose DNA methylation in cord blood was nominally significantly associated with prenatal opioid exposure. This was a CpG site that was initially identified in the BBC study in cord blood, and while the magnitude of methylation difference was smaller in our study, the direction of methylation change was the same.

Cg14303187 is annotated to the *SHC3* gene. This gene is predicted to be involved in glutamatergic synaptic transmission, as well as learning and memory (Lee et al., 2012; Park et al., 2023). Additionally, it has recently been identified as a susceptibility gene for schizophrenia (Lv et al., 2020). Prenatal opioid exposure has been associated with adverse neurodevelopmental outcomes in infants and children, including neonatal opioid withdrawal syndrome, impaired neuromotor, psychomotor, mental, and language development in infancy, and increased risk of attention problems later in life (Conradt et al., 2019). The mechanisms responsible, however, are not fully characterized. Future studies should focus on comprehensively characterizing the effects of prenatal opioid exposure on the epigenome in multiple tissues at birth to determine underlying biological networks or pathways that might be impacted. This could illuminate the mechanisms at play.

The second objective was to test whether prenatal opioid exposure was associated with accelerated or decelerated gestational epigenetic age. Epigenetic age is an emerging biomarker of an individual’s biological or physiological age, and studies have demonstrated that exposures and lifestyle factors are associated with deviations in one’s epigenetic age from their chronologic age (Dhingra et al., 2018; Ladd-Acosta et al., 2023; Song et al., 2022). To our knowledge, how prenatal opioid exposure might impact this biological aging pathway had not previously been explored; however, it is of particular interest given that prenatal opioid exposure has been associated with being born prematurely. In the present study, we did not detect any significant associations between prenatal opioid exposure and epigenetic age at birth. However, our sample sizes were small so this analysis should be repeated in a larger sample size to confirm if there is truly an absence of this association.

There are some limitations to the present study. First, as mentioned above, the sample sizes for opioid exposure were small. This could have hindered our ability to detect meaningful associations in both our CpG site-specific, and epigenetic age analyses, and limited our ability to identify significant findings after adjusting for multiple comparisons. Further, this subset of cohorts is not fully representative of ECHO as a whole, and future work should revisit and explore this question as additional DNAm samples become available. Second, there are well-established sex differences in the vulnerability of the developing embryo/fetus to *in utero* exposures, with individuals assigned male at birth being the more susceptible sex (Bale, 2016; Hodes & Epperson, 2019). However, we were not powered enough to stratify these analyses by sex, which may have limited our ability to detect any meaningful sex differences in the response of the epigenome to prenatal opioid exposure. Third, we focused our analyses primarily on cord blood and blood spots and thus we may have missed meaningful associations between prenatal opioid exposure and epigenetic changes in other tissues in ECHO such as placenta. Fourth, in our site-specific analyses, we were only powered to investigate a handful of CpG sites. Future studies that have large enough sample sizes should take an epigenome-wide approach to detect all possible meaningful associations between prenatal opioid exposure and DNA methylation changes at birth, and to discern biological pathways that might be affected or implicated in mediating exposure–outcome relationships. Lastly, maternal opioid use during pregnancy was a combination of medical record abstraction and self-reported measures, the latter of which could be subjected to recall and/or desirability bias.

The limitations of the present study are balanced by its strengths. This work is novel in its assessment of the relationship between prenatal opioid exposure and epigenetic age at birth. A further strength of this epigenetic age analysis was the examination of both extrinsic and intrinsic epigenetic age acceleration changes, the latter of which enabled us to control biologically relevant covariates like cell composition. More broadly, we included a robust set of harmonized covariate data in our analyses. We leveraged harmonized data from three cohorts for our CpG-specific analyses, and eight cohorts for the epigenetic age analyses, from a nationally representative US children’s cohort. This provides the necessary representation of both sociodemographic and geographic variability that is present across the country.

In this cohort study, we leveraged the robust resources available to us through the ECHO program to investigate the effects of prenatal opioid exposure on locus-specific DNA methylation changes and epigenetic age changes at birth. We identified a single CpG site from cord blood whose methylation was associated with prenatal opioid exposure. We did not detect any significant associations between exposure and epigenetic age at birth. Future studies require larger samples of exposed individuals to enable more robust investigation into the biological pathways and putative mechanisms impacted by prenatal opioid exposure. This is especially critical as prenatal exposure to opioids is rising drastically across the US.

## Supplementary Material

Supplementary Material

## Figures and Tables

**Table 1.** Descriptive statistics for ECHO participants across eligible cohorts – CpG site specific analytic population.

	Singe CpG Site Analytic Population Demographics		
	No Opioid Exposure	Yes Opioid Exposure	*p*-value

	(*n* = 360)	(*n* = 25)	
*Maternal Age*			
Mean (SD)	32.4 (4.91)	34.6 (5.38)	.06
Median [Min, Max]	32.0 [18.0, 45.0]	34.0 [25.0, 45.0]	
*Maternal Alcohol Use During Pregnancy*			
No	268 (74.4%)	19 (76.0%)	.89
Yes	70 (19.4%)	<10^a^	
Missing	22 (6.1%)	<5^a^	
*Maternal Tobacco Use During Pregnancy*			
No	348 (96.7%)	23 (92.0%)	1.00
Yes	<10^a^	<5^a^	
Missing	<5^a^	<5^a^	
*Maternal Marijuana Use During Pregnancy*			
No	343 (95.3%)	23 (92.0%)	.58
Yes	13 (3.6%)	<5^a^	
Missing	<5^a^	<5^a^	
*Maternal Race*			
White	295 (81.9%)	22 (88.0%)	.80
Black	27 (7.5%)	<5^a^	
Asian	12 (3.3%)	<5^a^	
Multiple Race	<10^a^	<5^a^	
Other	<10^a^	<5^a^	
Missing	14 (3.9%)	<5^a^	
*Maternal Ethnicity*			
Non-Hispanic	315 (87.5%)	23 (92.0%)	1.00
Hispanic	30 (8.3%)	<5^a^	
Missing	15 (4.2%)	<5^a^	
*Maternal Depression T-Score*			
Mean (SD)	50.3 (8.26)	50.2 (9.18)	.96
Median [Min, Max]	49.5 [33.0, 73.2]	51.2 [33.0, 63.5]	
Missing	133 (36.9%)	<5^a^	
*Maternal Education*			
Less Than High School	<10^a^	<5^a^	.52
High School or GED	24 (6.7%)	<5^a^	
Some College, No Degree, and Above	307 (85.3%)	22 (88.0%)	
Missing	22 (6.1%)	<5^a^	
*Child Sex*			
male	164 (45.6%)	14 (56.0%)	.42
female	196 (54.4%)	11 (44.0%)	
*Gestational Age (weeks)*			
Mean (SD)	39.4 (1.51)	39.3 (1.21)	.78
Median [Min, Max]	40.0 [30.0, 42.0]	39.0 [37.0, 41.0]	

**Table 2.** Descriptive statistics for ECHO participants across eligible cohorts – epigenetic age analytic population.

	Epigenetic Age Analytic Population Demographics		
	No Opioid Exposure (*n* = 687)	Yes Opioid Exposure (*n* = 29)	*p*-value
*Maternal Age*			
Mean (SD)	30.8 (5.50)	33.1 (6.23)	.06
Median [Min, Max]	31.0 [18.0, 46.0]	33.0 [20.0, 45.0]	
*Maternal Alcohol Use During Pregnancy*			
No	551 (80.2%)	21 (72.4%)	.12
Yes	97 (14.1%)	<10^a^	
Missing	39 (5.7%)	<5^a^	
*Maternal Tobacco Use During Pregnancy*			
No	649 (94.5%)	25 (86.2%)	.32
Yes	32 (4.7%)	<5^a^	
Missing	<10^a^	<5^a^	
*Maternal Marijuana Use During Pregnancy*			
No	459 (66.8%)	26 (89.7%)	.68
Yes	32 (4.7%)	<5^a^	
Missing	196 (28.5%)	<5^a^	
*Maternal Race*			
White	469 (68.3%)	24 (82.8%)	.14
Black	139 (20.2%)	<5^a^	
Asian	21 (3.1%)	<5^a^	
Multiple Race	14 (2.0%)	<5^a^	
Other	25 (3.6%)	<5^a^	
Missing	19 (2.8%)	<5^a^	
*Maternal Ethnicity*			
Non-Hispanic	569 (82.8%)	26 (89.7%)	.65
Hispanic	102 (14.8%)	<5^a^	
Missing	16 (2.3%)	<5^a^	
*Maternal Depression T-Score*			
Mean (SD)	50.8 (7.65)	50.3 (8.87)	.79
Median [Min, Max]	51.2 [33.0, 76.8]	51.2 [33.0, 63.5]	
Missing	168 (24.5%)	<5^a^	
*Maternal Education*			
Less Than High School	47 (6.8%)	<5^a^	.33
High School or GED	121 (17.6%)	<10^a^	
Some College, No Degree, and Above	496 (72.2%)	23 (79.3%)	
Missing	23 (3.3%)	<5^a^	
*Child Sex*			
male	318 (46.3%)	16 (55.2%)	.45
female	369 (53.7%)	13 (44.8%)	
*Gestational Age (weeks)*			
Mean (SD)	39.1 (1.53)	39.1 (1.30)	.80
Median [Min, Max]	39.0 [30.0, 42.0]	39.0 [36.0, 41.0]	

**Table 3.** Comparison of DNA methylation at loci of interest in ECHO to methylation changes in original studies.

cgID	Chromosome	Geneic location	Relationship to nearest CG Island	450K Gene Annotation	Methylation difference in *original BBC* study	Methylation difference in *present* study	Concordance across studies in direction of methylation change (yes/no)
cg06872138	5	body	N_Shelf	*ETF1*	−7.40%	−1.00%	yes
cg14303187	9	body	–	*SHC3*	−7.00%	−1.30%	yes
cg04382374	22	body	–	*FAM19A5*	−3.96%	0.00%	no
cg01050810	6	–	–	–	−3.87%	0.50%	no
cg27408345	10	TSS200; TSS1500	N_Shore	*PDCD4*	−3.85%	0.60%	no
cg17390675	7	–	N_Shelf	–	−3.78%	0.60%	no
cg03024860	9	body	N_Shore	*VAV2*	−3.62%	−0.50%	yes
cg08233909	3	TSS200;5’UTR	–	*VEPH1*	−3.54%	0.00%	no
cg09147586	22	body	N_Shelf	*PANX2*	−3.54%	−0.30%	yes
cg03717994	11	–	–	–	−3.44%	0.10%	no
cg25106172	11	body	S_Shelf	*LOC100130987*	−3.20%	0.10%	no
cg24004959	3	–	–	–	−2.90%	0.00%	no
cg11452329	6	–	–	–	−2.85%	0.40%	no
cg26273310	21	body	–	*SIM2*	−2.66%	0.00%	no
cg16890600	5	–	Island	*LOC100289230*	7.00%	−0.30%	no

**Table 4.** Multivariable results for the association between prenatal opioid exposure and cg14303187 DNA methylation.

Model	Beta	*p*	Lower CI	Upper CI
cg14303187 crude	−0.013	.01	−0.023	−0.0027
cg14303187 adjusted	−0.012	.02	−0.023	−0.002

**Table 5.** Multivariable results for the association between prenatal opioid exposure and gestational epigenetic age acceleration.

Model	Beta	*p*-value	Lower CI	Upper CI
Extrinsic age acceleration				
crude	0.01	.96	−0.55	0.57
adjusted	0.02	.94	−0.54	0.59
Intrinsic age acceleration				
crude	0.28	.29	−0.24	0.81
adjusted	0.25	.35	−0.28	0.78

## Data Availability

Data is available from authors upon request.
