# Supplementary material for: Prenatal opioid exposure and the early life epigenome: results from ECHO
Source: J Subst Use. Author manuscript; Available in PMC 2025 Jun 30. (PMC12208659; doi:10.1080/14659891.2024.2356569)
Supplement: Supplementary Material [file NIHMS2002235-supplement-Supplementary_Material.zip › ECHO_EC0589_SuppFig1.pptx]

## Slide 1
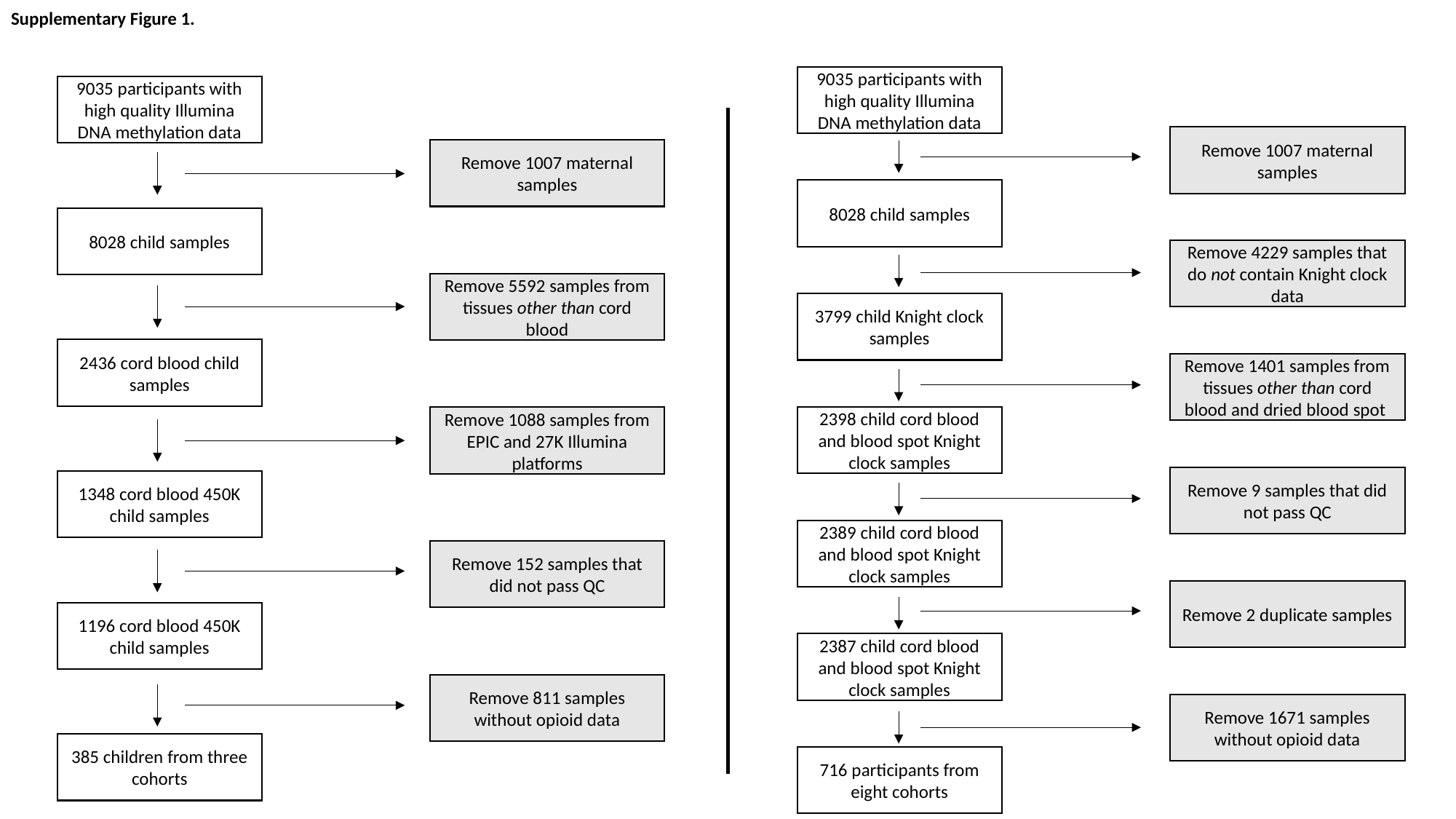

Supplementary Figure 1.
9035 participants with high quality Illumina DNA methylation data
9035 participants with high quality Illumina DNA methylation data
Remove 1007 maternal samples
Remove 1007 maternal samples
8028 child samples
8028 child samples
Remove 4229 samples that do not contain Knight clock data
Remove 5592 samples from tissues other than cord blood
3799 child Knight clock samples
2436 cord blood child samples
Remove 1401 samples from tissues other than cord blood and dried blood spot
2398 child cord blood and blood spot Knight clock samples
Remove 1088 samples from EPIC and 27K Illumina platforms
Remove 9 samples that did not pass QC
1348 cord blood 450K child samples
2389 child cord blood and blood spot Knight clock samples
Remove 152 samples that did not pass QC
Remove 2 duplicate samples
1196 cord blood 450K child samples
2387 child cord blood and blood spot Knight clock samples
Remove 811 samples without opioid data
Remove 1671 samples without opioid data
385 children from three cohorts
716 participants from eight cohorts
