# Supplementary material for: Prenatal opioid exposure and the early life epigenome: results from ECHO
Source: J Subst Use. Author manuscript; Available in PMC 2025 Jun 30. (PMC12208659; doi:10.1080/14659891.2024.2356569)
Supplement: Supplementary Material [file NIHMS2002235-supplement-Supplementary_Material.zip › ECHO_EC0589_SuppFig4.pptx]

## Slide 1
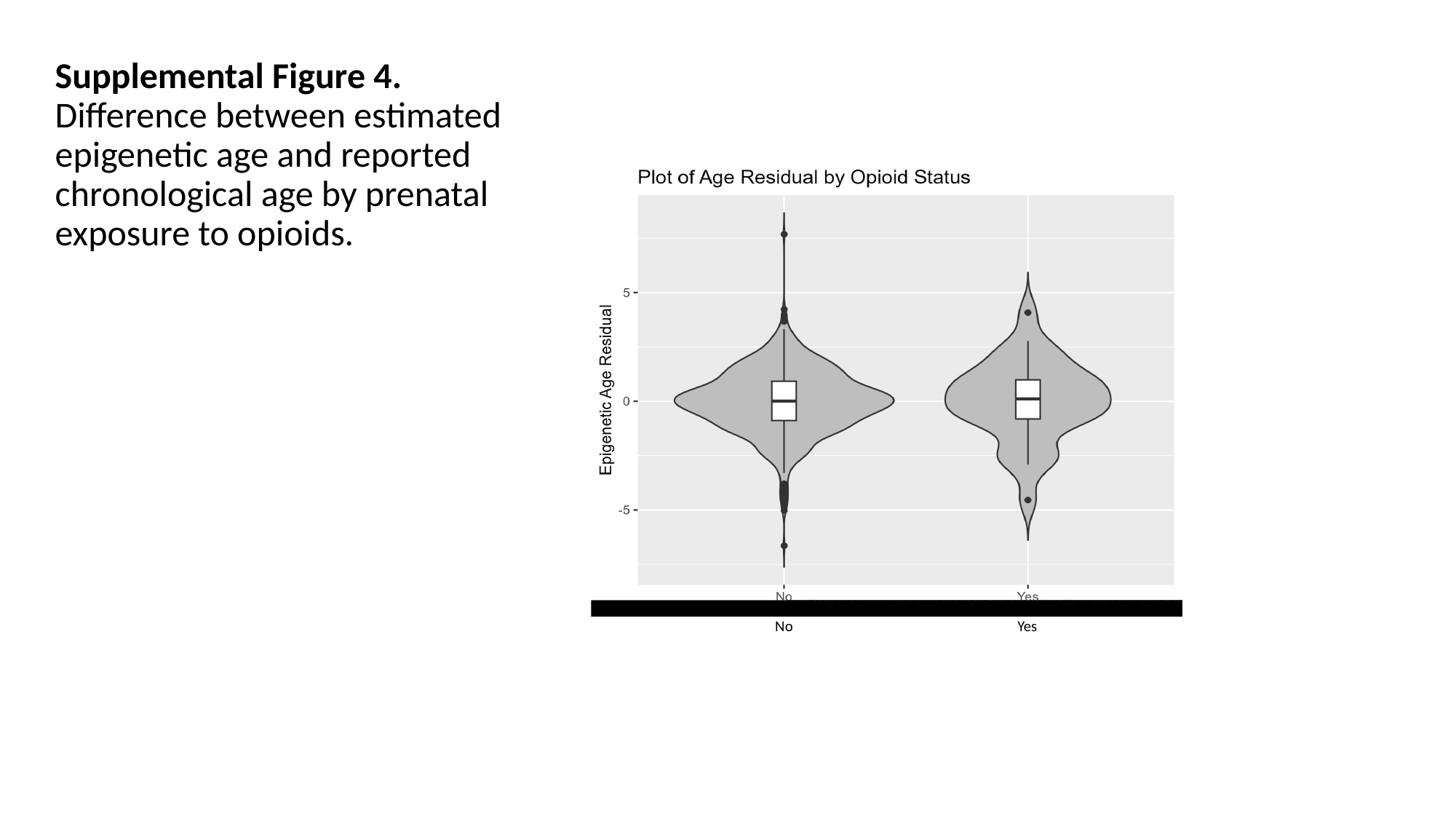

Supplemental Figure 4. Difference between estimated epigenetic age and reported chronological age by prenatal exposure to opioids.
Yes
No
