# Supplementary material for: Prenatal opioid exposure and the early life epigenome: results from ECHO
Source: J Subst Use. Author manuscript; Available in PMC 2025 Jun 30. (PMC12208659; doi:10.1080/14659891.2024.2356569)
Supplement: Supplementary Material [file NIHMS2002235-supplement-Supplementary_Material.zip › title page 2023-0280.R1.docx]

**Prenatal opioid exposure and the early life epigenome: results from ECHO.**

Rose Schrott^1,2^, Henri Garrison-Desany^3^, Lyndsay Avalos^4^, Carrie V. Breton^5^, Dana M. Dabelea^6^, Karen Derefinko^7^, Anne Dunlop^8^, Fang Fang^9^, Abigail Gaylord^10^, Torie Grant^11^, Marie-France Hivert^12^, Margaret R. Karagas^13^, Anna K. Knight^8^, Barry Lester^14^, Kristen Lyall^15^, Cindy McEvoy^16^, Ruby Nguyen^17^, Grier Page^9^, Alison Paquette^18^, Douglas Ruden^19^, Lyndsey E Shorey-Kendrick^20^, Alicia K. Smith^9^, Eliot Spindel^20^, Heather E. Volk^1,2^, Christine Ladd-Acosta^2,21,#^ on behalf of program collaborators for Environmental influences on Child Health Outcomes (ECHO) *

*See Acknowledgments for full listing of collaborators

^1^Wendy Klag Center for Autism and Developmental Disabilities, Johns Hopkins Bloomberg School of Public Health, Baltimore, MD, USA

^2^Department of Mental Health, Johns Hopkins Bloomberg School of Public Health, Baltimore, MD, USA

^3^ Department of Social and Behavioral Sciences, Harvard T. H. Chan School of Public Health, Boston MA

^4^ Division of Research, Kaiser Permanente Northern California, Oakland, CA, USA

^5^ Department of Preventive Medicine, Keck School of Medicine, University of Southern California, Los Angeles, CA, USA

^6^ Lifecourse Epidemiology of Adiposity and Diabetes Center, University of Colorado Anschutz Medical Campus, Aurora, Colorado, USA

^7^ Department of Preventive Medicine and Department of Pharmacology, Addiction Science, and Toxicology, The University of Tennessee Health Science Center, Memphis, TN, USA

^8^ Department of Gynecology and Obstetrics, Emory University School of Medicine, Atlanta, Georgia, USA.

^9^ RTI International, Research Triangle Park, North Carolina, Durham, USA

^10^ Department of Environmental Health Sciences, Columbia Mailman School of Public Health, New York, NY, USA

^11^ Department of Medicine and Department of Pediatrics, Johns Hopkins University School of Medicine

Baltimore, MD, USA

^12^ Department of Population Medicine, Harvard Medical School and Harvard Pilgrim Health Care Institute, Boston, MA, 02215, USA

^13^ Department of Epidemiology, Geisel School of Medicine, Dartmouth College, Hanover, New Hampshire, USA

^14^ Departments of Psychiatry and Pediatrics, Alpert Medical School of Brown University, Providence, RI, USA

^15^ AJ Drexel Autism Institute, Drexel University, Philadelphia, Pennsylvania, USA

^16^ Department of Pediatrics, Oregon Health and Science University, Portland, OR, USA

^17^ Division of Epidemiology and Community Health, University of Minnesota, Minneapolis, MN, USA

^18^ Department of Pediatrics, School of Medicine, University of Washington, Seattle, WA, USA; Seattle Children's Research Institute, Seattle, WA, USA

^19^ Department of Obstetrics and Gynecology, Wayne State University, Detroit, Michigan

^20^ Division of Neuroscience, Oregon National Primate Research Center, Oregon Health & Science University, Beaverton, Oregon

^21^ Department of Epidemiology, Johns Hopkins Bloomberg School of Public Health, Baltimore, MD, USA.

^#^ Corresponding author ([claddac1@jhu.edu](mailto:claddac1@jhu.edu))
