# Supplementary figures and images for: Prenatal opioid exposure and the early life epigenome: results from ECHO
Source: J Subst Use. Author manuscript; Available in PMC 2025 Jun 30. (PMC12208659; doi:10.1080/14659891.2024.2356569)

## Slide 1
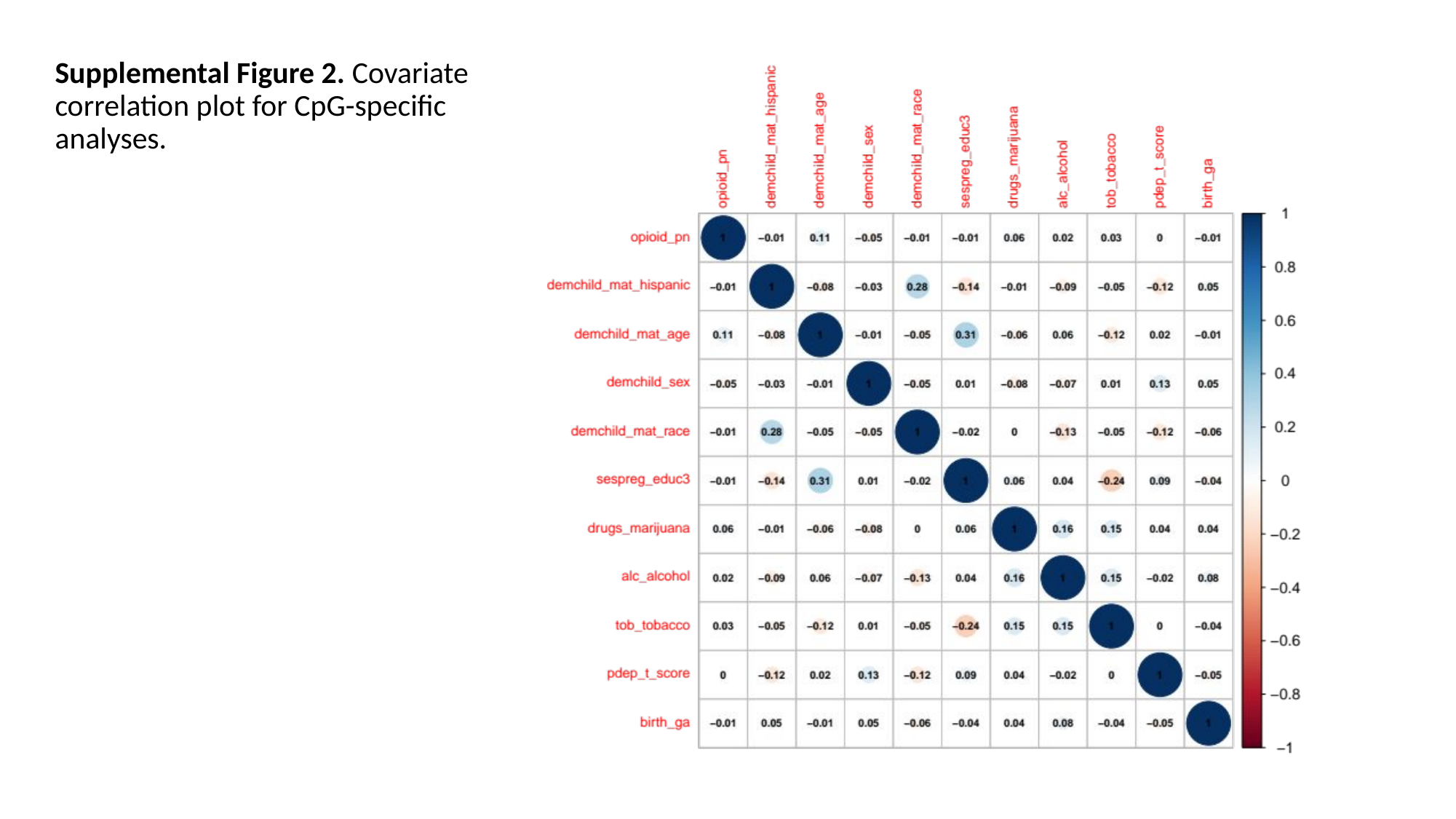

Supplemental Figure 2. Covariate correlation plot for CpG-specific analyses.

Supplement: Supplementary Material [file NIHMS2002235-supplement-Supplementary_Material.zip › ECHO_EC0589_SuppFig2.pptx]

## Slide 1
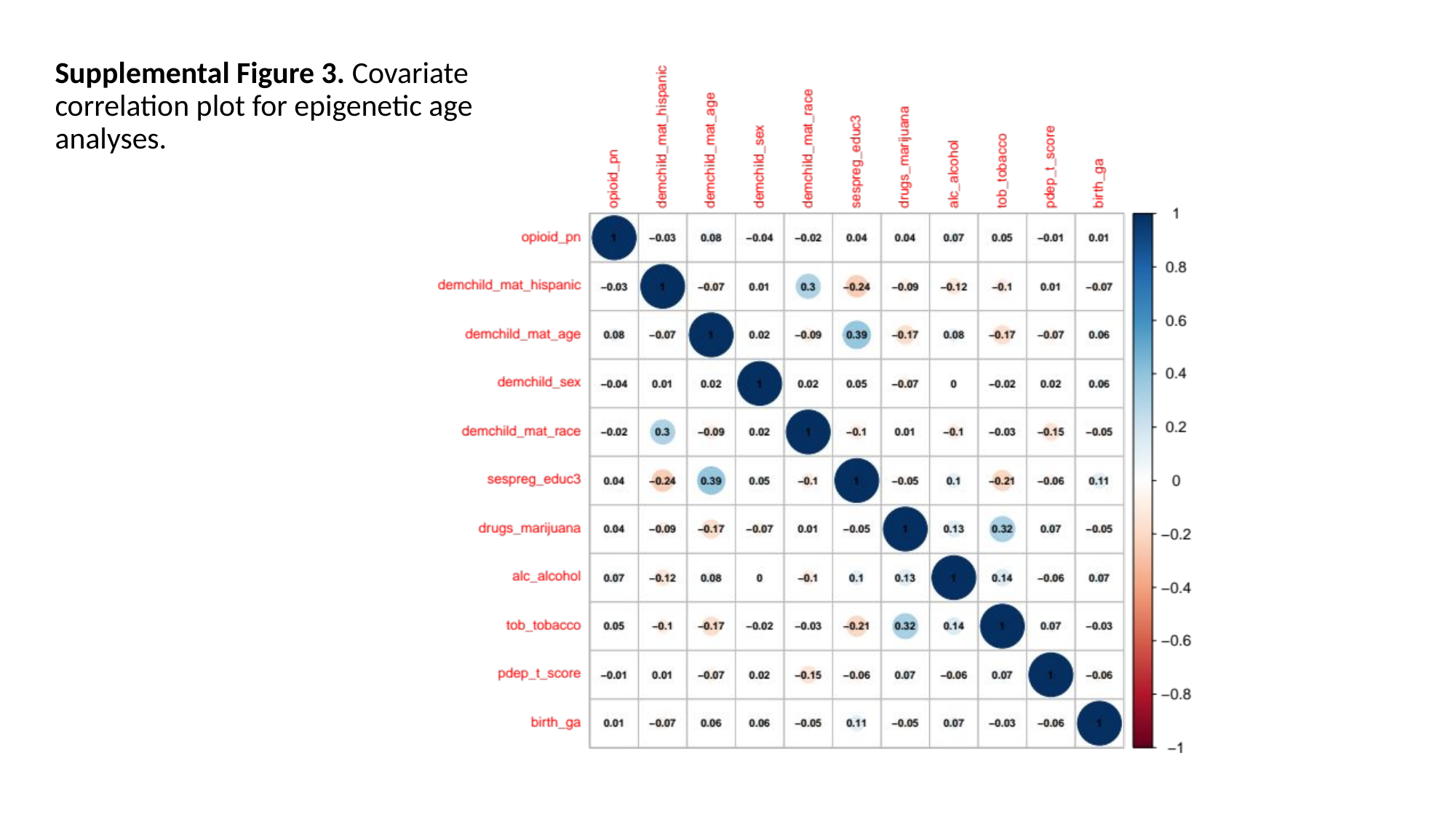

Supplemental Figure 3. Covariate correlation plot for epigenetic age analyses.

Supplement: Supplementary Material [file NIHMS2002235-supplement-Supplementary_Material.zip › ECHO_EC0589_SuppFig3.pptx]
